# Inverse association between insulin resistance and gait speed in nondiabetic older men: results from the U.S. National Health and Nutrition Examination Survey (NHANES) 1999-2002

**DOI:** 10.1186/1471-2318-9-49

**Published:** 2009-11-19

**Authors:** Chen-Ko Kuo, Lian-Yu Lin, Yau-Hua Yu, Kuan-Han Wu, Hsu-Ko Kuo

**Affiliations:** 1 Department of Emergency Medicine, Chang-Gung Memorial Hospital-Kaohsiung Medical Center, No.123, Dapi Rd., Niaosong Township, Kaohsiung County 833, Taiwan; 2 Department of Emergency Medicine, Chang Gung University College of Medicine, 259 Wen-Hwa 1st Road, Kwei-Shan Taoyuan 333, Taiwan; 3 Department of Internal Medicine, National Taiwan University Hospital, 7 Chun-Shan South Road, Taipei 100, Taiwan; 4 Department of Geriatrics and Gerontology, National Taiwan University Hospital, 7 Chun-Shan South Road, Taipei 100, Taiwan; 5 School of Dentistry, National Yang Ming University, No.155, Sec. 2, Linong St., Beitou Dist., Taipei 112, Taiwan; 6 Department of Dentistry, Taipei Veterans General Hospital, No. 201, Sec. 2, Shih-Pai Road, Taipei 112, Taiwan; 7 Department of Medical Research and Development, Taipei Veterans General Hospital, No. 201, Sec. 2, Shih-Pai Road, Taipei 112, Taiwan; 8 Division of Gerontology Research, Institute of Population Health Sciences, National Health Research Institutes, 35 Keyan Road, Zhunan, Miaoli County 350, Taiwan

## Abstract

**Background:**

Recent studies have revealed the associations between insulin resistance (IR) and geriatric conditions such as frailty and cognitive impairment. However, little is known about the relation of IR to physical impairment and limitation in the aging process, eg. slow gait speed and poor muscle strength. The aim of this study is to determine the effect of IR in performance-based physical function, specifically gait speed and leg strength, among nondiabetic older adults.

**Methods:**

Cross-sectional data were from the population-based National Health and Nutrition Examination Survey (1999-2002). A total of 1168 nondiabetic adults (≥ 50 years) with nonmissing values in fasting measures of insulin and glucose, habitual gait speed (HGS), and leg strength were analyzed. IR was assessed by homeostasis model assessment (HOMA-IR), whereas HGS and peak leg strength by the 20-foot timed walk test and an isokinetic dynamometer, respectively. We used multiple linear regression to examine the association between IR and performance-based physical function.

**Results:**

IR was inversely associated with gait speed among the men. After adjusting demographics, body mass index, alcohol consumption, smoking status, chronic co-morbidities, and markers of nutrition and cardiovascular risk, each increment of 1 standard deviation in the HOMA-IR level was associated with a 0.04 m/sec decrease (p = 0.003) in the HGS in men. We did not find such association among the women. The IR-HGS association was not changed after further adjustment of leg strength. Last, HOMA-IR was not demonstrated in association with peak leg strength.

**Conclusion:**

IR is inversely associated with HGS among older men without diabetes. The results suggest that IR, an important indicator of gait function among men, could be further investigated as an intervenable target to prevent walking limitation.

## Background

Recently, increasing research effort has been focused on the role of IR in age-related conditions or geriatric syndromes. The Italian InCHIANTI study, by examining community-dwelling elderly population, suggested that IR is associated with cognitive impairment as evidenced by poor performance in Trail Making Test and Mini-Mental State Examination [1,2]. By following 3141 community-dwelling adults for 10 years, Barzilay and colleagues from the Cardiovascular Health Study demonstrated that IR is associated with incident frailty [3], a composite outcome consisting of involuntary weight loss, exhaustion, low physical activity, slowness, and weakness. With life expectancy reaching its historic pinnacle, decline in physical performance and the resultant late-life disability have become common features in an aging society [4-9]. However, data examining the association between IR and physical function are relatively sparse. We hypothesized that IR is inversely associated with physical function in community-dwelling elders. We sought to test the hypotheses by analyzing data from the NHANES 1999-2002.

## Methods

### Study Design and Population

The NHANES, a population-based survey, used a stratified, multistage, and cluster sampling design to obtain a representative sample of the noninstitutionalized U.S. civilian population. NHANES consists of a detailed home interview and a health examination conducted in a mobile examination center (MEC). Datasets, Survey Operations Manuals, Consent Documents, and Brochures of the NHANES 1999-2002 are available on the NHANES website [10,11].

A subsample of 2052 participants aged 50 years and older were randomly assigned to a morning session and had complete information of fasting plasma glucose and serum insulin. Individuals who had diabetes (physician-diagnosed history, fasting plasma glucose ≥ 126 mg/dL, or current use of diabetes medication; n = 434) or with missing values in the timed walking and isokinetic leg strength tests because of safety concerns (recent chest or abdominal surgery; heart attack in the past 6 weeks; brain aneurysm or stroke; current neck or back pain; difficulty in bending or straightening right knee; or right knee or right hip replacement) or any administrative, communicative, or technical problems (n = 450) were excluded from the analysis, leaving 1168 participants as the analytic sample. Among the final sample, median values of C-reactive protein (0.27 mg/dL, n = 1), vitamin B_12 _(465.5 pg/mL, n = 2), folate (15.1 ng/mL, n = 3), total cholesterol (212 mg/dL, n = 2), cotinine (0.05 ng/mL, n = 24), as well as body mass index (27.12 kg/m2, n = 12) were used to impute missing values for these variables, all of which were used in the analysis.

### Measures of IR

A blood sample was collected following an overnight fast (≥ 8 h) in participants who were assigned to a physical examination during a morning session. Plasma glucose level was measured with a hexokinase enzymatic reference method (COBRAS MIRA; Roche Diagnostics, Indianapolis, IN) and serum insulin level by a radio-immunoassay (Pharmacia Diagnostics, Uppsala, Sweden). Homeostasis model assessment of IR (HOMA-IR), an estimate of IR commonly applied in large epidemiological studies, was used to evaluate IR using the following formula: fasting serum insulin (μU/mL) × fasting plasma glucose (mmol/L)/22.5 [12].

### Measures of habitual gait speed and isokinetic leg strength

Habitual gait speed (HGS) was measured in the MEC according to the NHANES standardized protocol. A 20 feet long test tract area was set up in a corridor of the MEC. The 20-foot walk was timed using a hand-held stopwatch. The 20-foot timed walk test was performed at the participant's usual pace. Use of a walker or cane was allowed if needed. HGS was calculated as walking distance (20 feet = 6.15 m) divided by time (second). Maximal right knee extensor force (Newton) was measured at an angular velocity of 60 degree/second by a Kinetic Communicator isokinetic dynamometer (Chattecx Corp., Chattanooga, TN).

### Covariates

Age, gender, and race/ethnicity were obtained by self-report. Co-morbidities including heart disease (defined as a history of myocardial infarction, coronary heart disease, congestive heart failure or angina), chronic lung disease (defined as chronic bronchitis or emphysema), and arthritis were ascertained by self-report questionnaires. Alcohol intake was determined by the questionnaire "In any one year, have you had at least 12 drinks of any type of alcohol beverage?" and was dichotomized. Exposure to tobacco smoke increases the concentration of nicotine in the blood, and nicotine is highly specific for such exposure. Cotinine is a major metabolite of nicotine. Using their serum cotinine concentrations (ng/mL), we classified smoking status of participants in four groups: nonsmoker (<14), light smoker (14-99), moderate smoker (100-199), and heavy smoker (≥ 200) [13]. Three and sometimes 4 blood pressure (BP) determinations were taken using a mercury sphygmomanometer by a NHANES physician. BP was measured in the right arm unless specific conditions prohibit the use of the right arm. Averaged systolic and diastolic BPs were obtained. The presence of hypertension was defined by a self-report doctor's diagnosis, the use of anti-hypertensive medications, or averaged blood pressure greater than 140/90 mmHg. Body mass index (BMI), calculated as weight in kilograms divided by the square of height in meters, was categorized according to the National Institutes of Health obesity standards: <18.5 = underweight, 18.5-24.9 = normal weight, 25.0-29.9 = overweight, and >30 = obese [14]. C-Reactive protein (CRP) was quantified by utilizing latex-enhanced nephelometry with a Behring Nephelometer Analyzer System (Behring Diagnostics, Frankfurt, Germany). Plasma homocysteine was measured by the Abbott homocysteine assay (Abbott Park, IL, USA), a fully automated fluorescence polarization immunoassay (FPIA) method.

### Analysis

Data were analyzed using Stata 9.0 (Stata, College Station, TX) specialized for complex survey data. We used stratum and primary sampling unit (PSU) variables required for variance estimation. In order to account for clustering and stratification of the multi-stage NHANES sampling design, we incorporated appropriate sampling weight in all analyses in order to obtain point estimates and standard errors (SEs) applicable to the U.S. population. In the NHANES 1999-2002, separate 4-year fasting weights were created to reflect the additional stage of sampling and the additional nonresponse for the subsample of fasting participants assigned to the morning session. These 4-year fasting weights were used for data analysis.

Characteristics of the study population, including means and SEs values for continuous variables and percentages for categorical variables, were calculated with correction for the survey design in both men and women. Stratified by sex, weighted linear regression analysis was used to examine the relation of HOMA-IR to performance-based physical measures, namely leg strength and HGS. The distributions of HOMA-IR in both men and women were right-skewed. Therefore, we used natural-log-transformed values, which provided the best-fitting model for analysis in which the HOMA-IR were treated as a continuous variable. For men, standard-deviation scores of HOMA-IR were obtained from the formula (X_i_-X_m_) ÷ SD, where X_i _was the natural-log-transformed HOMA-IR in the individual male subject, X_m _the mean natural-log-transformed HOMA-IR in the male subjects, and SD the standard deviation of the natural-log-transformed HOMA-IR in the male subjects. The standard-deviation scores of HOMA-IR in women were obtained from the same formula. This calculation allowed us to determine the change in the gait speed and leg strength for each increment of 1SD in the natural-log-transformed HOMA-IR. The relations of HOMA-IR to leg strength and gait speed were also evaluated with a quartile-based analysis by dividing HOMA-IR levels into quartiles with subjects in the lowest quartile as the reference group. We used an extended-model approach for covariates adjustments: Model 1 = age, race, BMI, smoking status, alcohol consumption, and use of walking device; Model 2 = Model 1 + chronic diseases (hypertension, chronic lung disease, heart diseases, and arthritis); Model 3 = Model 2 + markers of nutrition and cardiovascular risk (natural-log-transformed levels of folate, vitamin B_12_, total cholesterol, homocysteine and CRP). Given the fact that muscle strength has been shown to be an important correlate for walking speed among community-dwelling older adults [15], we additionally controlled for leg strength in the association between HOMA-IR and HGS (Model 4) in order to observe possible change of association.

## Results

Table 1 lists the characteristics of the participants according to sex. The mean age among the 566 men was 61.8 years (age range 50 to 85 years), and among the 602 women it was 62.4 years (age range 50 to 85 years). The men had higher fasting plasma glucose, serum insulin, HOMA-IR, peak leg strength, as well as a higher prevalence of heart disease compared to women. The presence of hypertension, chronic lung disease, and arthritis was more common in women than in men. The men smoked more than the women and consumed more alcohol. The cut-off values for HOMA-IR quartiles among the men were: quartile 1 (<1.80), quartile 2 (1.80-2.51), quartile 3 (2.52-3.69), and quartile 4 (>3.69); while among the women they were quartile 1 (<1.48), quartile 2 (1.48-2.28), quartile 3 (2.29-3.52), and quartile 4 (>3.52). Men were comparable to women in terms of age, BMI, race, and HGS.

**Table 1:** Characteristics of Study Participants NHANES 1999-2002 (N = 1168)

Characteristics	Men (N = 566)	Women (N = 602)	P value
**Continuous variables, Mean (SE)**			
Age (year)	61.8 (0.4)	62.4 (0.3)	0.202
Body mass index (kg/m2)	27.9 (0.3)	27.7 (0.3)	0.752
Fasting plasma glucose (mg/dL)	101.0 (0.5)	97.4 (0.5)	<0.001
Serum insulin (μU/mL)	11.72 (0.4)	10.52 (0.2)	0.001
Insulin resistance (HOMA-IR)	2.96 (0.1)	2.59 (0.1)	<0.001
Peak leg strength (Newton)	439 (6.2)	299 (4.3)	<0.001
Habitual gait speed (m/sec)	1.090 (0.013)	1.083 (0.011)	0.620
**Categorical variables, N (weighted %)**			
Race			0.427
Mexican American	104 (3.2)	114 (3.0)	
Other Hispanic	22 (3.5)	28 (5.2)	
Non-Hispanic White	367 (85.9)	372 (83.5)	
Non-Hispanic Black	62 (4.8)	77 (5.8)	
All others	11 (2.6)	11 (2.5)	
Hypertension	294 (47.1)	387 (58.9)	0.001
Heart diseases	73 (12.4)	50 (7.5)	0.017
Chronic lung disease	30 (4.4)	58 (11.4)	0.001
Arthritis	184 (32.5)	278 (46.7)	<0.001
Smoking status			0.001
Nonsmoker	429 (73.3)	514 (84.2)	
Light smoker	22 (3.3)	14 (2.3)	
Moderate smoker	39 (7.3)	35 (6.5)	
Heavy smoker	76 (16.1)	39 (7.0)	
Had > 12 alcohol drinks/year	464 (82.3)	314 (60.5)	<0.001

Values were expressed as mean (SEs) and number (weighted %)

**Figure 1:**
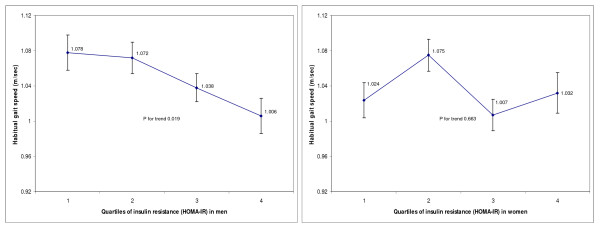
**Adjusted means of habitual gait speed v.s. quartiles of insulin resistance (HOMA-IR) in both men and women**.* * Means of habitual gait speed were adjusted for age, race, body mass index categories, smoking status, alcohol consumption, use of walking devices, co-morbidities (hypertension, chronic lung disease, heart disease, and arthritis), markers of nutrition and cardiovascular risk (natural-log-transformed levels of folate, vitamin B_12_, total cholesterol, homocysteine and C-reactive protein), and peak leg strength. Abbreviation: homeostasis model assessment of insulin resistance HOMA-IR.

HOMA-IR levels were inversely associated with HGS among the men. After adjustment for age, race, BMI categories, alcohol consumption, and use of walking devices, each increment of 1SD in the HOMA-IR level was associated with a 0.035 m/sec decrease (p = 0.007) in HGS (Table 2). Additional adjustment of covariates including chronic co-morbidities and markers of nutrition/cardiovascular risk (folate, vitamin B12, total cholesterol, homocysteine, and CRP) did not change the association among men (Model 2 and Model 3). In the full-adjusted model where peak leg strength was additionally adjusted (Model 4), the negative association between HOMA-IR and HGS among men remained (β coefficient -0.042, p = 0.001). We did not find any association between HOMA-IR and HGS among the women. We subsequently divided HOMA-IR levels into quartiles and showed that HGS for men in the highest HOMA-IR quartile were 0.066 m/sec less than that for men in the lowest quartile after adjustment for Model 1 covariates (significant trend across HOMA-IR quartiles with p = 0.035). Likewise, supplementary adjustment for additional covariates (Models 2 to Model 4) did not change the inverse association between HOMA-IR and HGS among the men in the quartile-based analyses. We did not find a clear trend between HOMA-IR quartiles and HGS among the women. Stratified by gender, adjusted means of HGS based on different HOMA-IR quartiles were obtained from the full-adjusted regression models (Model 4) and illustrated in the Figure 1. We did not find any association between HOMA-IR and peak leg strength in both men and women (Data not shown).

**Table 2:** Association between insulin resistance (HOMA-IR) and gait speed in men and women

	Models with HOMA-IR as a continuous variable							
	
	Men				Women			
		
		β* (SE)	P value			β* (SE)	P value	
Model 1		-0.035 (0.013)	0.007			-0.018 (0.013)	0.172	
Model 2		-0.033 (0.013)	0.010			-0.014 (0.013)	0.289	
Model 3		-0.040 (0.013)	0.003			-0.011 (0.013)	0.377	
Model 4		-0.042 (0.012)	0.001			-0.012 (0.013)	0.364	

	**Models with HOMA-IR by increasing quartiles**							
	
	**Men**				**Women**			
	
	**Quartile comparison**	**β†** **(SE)**	**P value**	**P for trend**	**Quartile comparison**	**β†** **(SE)**	**P value**	**P for trend**

Model 1	Q2 v.s. Q1	-0.013 (0.026)	0.616	0.035	Q2 v.s. Q1	0.049 (0.027)	0.072	0.446
	Q3 v.s. Q1	-0.043 (0.029)	0.141		Q3 v.s. Q1	-0.032 (0.031)	0.304	
	Q4 v.s. Q1	-0.066 (0.033)	0.046		Q4 v.s. Q1	-0.001 (0.035)	0.987	

Model 2	Q2 v.s. Q1	-0.010 (0.026)	0.711	0.056	Q2 v.s. Q1	0.052 (0.027)	0.057	0.521
	Q3 v.s. Q1	-0.039 (0.029)	0.177		Q3 v.s. Q1	-0.025 (0.030)	0.403	
	Q4 v.s. Q1	-0.058 (0.033)	0.078		Q4 v.s. Q1	0.004 (0.033)	0.905	

Model 3	Q2 v.s. Q1	-0.015 (0.027)	0.567	0.024	Q2 v.s. Q1	0.055 (0.025)	0.042	0.700
	Q3 v.s. Q1	-0.046 (0.030)	0.120		Q3 v.s. Q1	-0.020 (0.030)	0.504	
	Q4 v.s. Q1	-0.072 (0.033)	0.030		Q4 v.s. Q1	0.013 (0.033)	0.702	

Model 4	Q2 v.s. Q1	-0.006 (0.026)	0.786	0.019	Q2 v.s. Q1	0.051 (0.026)	0.054	0.663
	Q3 v.s. Q1	-0.039 (0.028)	0.167		Q3 v.s. Q1	-0.018 (0.030)	0.556	
	Q4 v.s. Q1	-0.072 (0.032)	0.024		Q4 v.s. Q1	0.008 (0.033)	0.807	

Adjusted covariates:

Model 1 = Age, race, body mass index categories, smoking status, alcohol consumption, and use of walking devices

Model 2 = Model 1 + co-morbidities (hypertension, chronic lung disease, heart disease, and arthritis)

Model 3 = Model 2 + markers of nutrition and cardiovascular risk (natural-log-transformed levels of folate, vitamin B_12_, total cholesterol, homocysteine and C-reactive protein).

Model 4 = Model 3 + peak leg strength

* Parameter estimates (β) can be interpreted as differences in mean gait speed (m/sec) for each increment of one standard deviation in the log transformed HOMA-IR among men (or women).

† Parameter estimates (β) can be interpreted as differences in mean gait speed (m/sec) compared male (or female) subjects in the 2^nd^, 3^rd^, and 4^th ^quartiles of HOMA-IR to those in the lowest quartile.

Abbreviations: homeostasis model assessment of insulin resistance, HOMA-IR; SE, standard error.

The cut-off values HOMA-IR quartiles among the men were: quartile 1 (<1.80), quartile 2 (1.80-2.51), quartile 3 (2.52-3.69), and quartile 4 (>3.69); while among the women the cut-off values were: quartile 1 (<1.48), quartile 2 (1.48-2.28), quartile 3 (2.29-3.52), and quartile 4 (>3.52).

## Discussion and conclusion

Among men without diabetes, there was an inverse association between IR and HGS. The relationship was independent of age, race, BMI, smoking status, alcohol consumption, co-morbidities, markers of nutrition and cardiovascular risk, and peak leg strength. Our findings support and extend previous studies examining the role of metabolic syndrome, a cluster of cardiovascular risk factors strongly associated with IR, in predicting the development of functional impairment. Supporting statements from Blazer and colleagues, by analyzing 1229 older adults from the Duke Established Populations for Epidemiologic Studies of the Elderly (EPESE), demonstrated that metabolic syndrome independently predicted mobility decline, as assessed by the ability to perform heavy housework unaided, walk up and down a flight of stairs unaided, and walk half a mile unaided[16]. Additionally, the population-based Sacramento Area Latino Study on Aging (SALSA) reinforced the fact that metabolic syndrome was associated with progressive limitations in mobility and strength by following 1606 Mexican Americans older adults for 3 years[17]. Okoro and colleagues, cross-sectionally examining the NHANES cohort, suggested that selected components of metabolic syndrome, specifically low HDL cholesterol and abdominal obesity, were associated with gait impairment[18]. Nevertheless, these studies may have weakness in internal validity because such important confounding factors as chronic inflammation [16-18], medical co-morbidities[16,18], and nutritional status [16-18] were not considered. Furthermore, the reports of functional impairment are self-reported in Blazer et al[16] and Blaum et al[17], thus imposing potential bias in outcome ascertainment. Although metabolic syndrome, a composite outcome defined by expert panel[19], has recently attracted research interest, the usefulness of metabolic syndrome as an actual pathophysiological basis of functional decline has been questioned[20]. On the other hand, IR has been proposed as a principal factor in initiating and perpetuating the pathologic manifestations of the metabolic syndrome[21]. Unfortunately, none of the above studies scrutinize the role of IR in functional status. **The cross-sectional relation of IR to muscle strength has been evaluated in two population-based studies of community dwellers that highlighted an inverse association between IR and muscle strength **[22-24]. **However, such studies used different confounding variables in their statistical models and one of the studies consisted of a cohort entirely made up of men**[22]**which may explain the discrepancies from our findings**. To our knowledge, this is the first report to describe the association between IR and performance-based physical function among older men and women by using a large group of geographically dispersed and ethnically diverse national population-based sample. Potential confounders were comprehensively considered and the role of leg strength in the association between IR and gait speed was assessed.

By acting as a cardiovascular risk factor, IR may cause cerebral atherosclerotic changes in the cerebral circulation [25], **thus contributing to the development of cerebral micro-angiopathy (ie, leukoaraiosis or cerebral small vessel disease). The lesions may disrupt the integrity of frontal-subcortical circuits, thus compromising gait function**. Cerebrovascular lesions thus provide a conceivable mechanism between IR and slow gait speed. Since the association between IR and gait speed varies across gender, there may be an effect modification of sex in the association. **The fact that men had a greater baseline prevalence of cardiovascular risk (such as heart disease and smoking) than women may justify why an inverse association between IR and gait speed is seen in men but not in women**. Further research efforts are needed to investigate possible mediating factors, such as differential coordinating processes in brain neural circuiting or sex hormones[26], in mediating the effect of IR on gait speed. Our results have clinical implications. IR, in addition to its metabolic and cardiovascular implications, may serve as an important indicator of walking function among older man. As such, IR may be a promising target for intervention to prevent decline of walking function in late life. Secondly, pharmaceutical interventions such as metformin and thiazolidinedione [27], as well as additional known strategies to effectively lower IR- healthy dietary pattern [28], regular exercise [29], or weight reduction [30]- may **additionally **improve walking function and prevent functional decline, especially for high-risk subjects.

Our study has potential limitations deserving comments. First, due to the cross-sectional design, causal relationship between IR and gait speed can not be established. The association between IR and gait speed could simply be epiphenomenon of aging and the association should be prospectively explored. Second, although we have comprehensively adjusted for such confounders as chronic co-morbidities, markers of nutrition, inflammation, and cardiovascular risk, as well as leg strength in the association of IR and gait speed, other important measures such as cognitive ability, symptoms of depression, markers of endothelial dysfunction, or evidence of cerebral small vessel disease are absent or incomplete in the NHANES 1999-2002 dataset.

In conclusion, a higher level of IR was associated with a slower gait speed among non-diabetic older men. The association does not exist in the women. We provided new information for the association between IR and gait speed among community-dwelling older adults where data currently do not exist.

## Competing interests

The authors declare that they have no competing interests.

## Authors' contributions

All authors critically reviewed the manuscript, read and approved the final manuscript.

## Pre-publication history

The pre-publication history for this paper can be accessed here:

http://www.biomedcentral.com/1471-2318/9/49/prepub
